# “Finding my own motivation” — A Mixed Methods Study of Exercise and Behaviour Change Support During Oncological Treatment

**DOI:** 10.1007/s12529-019-09809-z

**Published:** 2019-08-22

**Authors:** Anne-Sophie Mazzoni, Maria Carlsson, Sveinung Berntsen, Karin Nordin, Ingrid Demmelmaier

**Affiliations:** 1 Department of Public Health and Caring Sciences, Section of Lifestyle and Rehabilitation in Long-term Illness, Uppsala University, Uppsala, Sweden; 2 Department of Public Health, Sport and Nutrition, Faculty of Health and Sport Sciences, University of Agder, Kristiansand, Norway

**Keywords:** Behaviour change techniques, Cancer, Incentives, Physical activity, Qualitative research, Self-determination theory

## Abstract

**Background:**

Exercising during oncological treatment is beneficial but challenging for persons with cancer and may require strategies to increase motivation. Behaviour change support, including specific behaviour change techniques (BCTs), have been used to facilitate exercise in persons undergoing oncological treatment, but more detailed knowledge from an individual perspective is needed to inform clinical practice. The aims were to explore the motivational experiences of exercise combined with behaviour change support, and to describe how specific BCTs were valued among persons exercising during oncological treatment.

**Methods:**

A mixed-methods study was conducted using semi-structured interviews (*n* = 18) and a questionnaire (*n* = 229). Participants with breast, colorectal or prostate cancer who completed or dropped out of a six-month exercise programme during oncological treatment were included. The interviews were analysed with thematic analysis and the questionnaire with descriptive statistics (median and interquartile range).

**Results:**

The participants underwent a motivational process through the exercise programme. By experiencing ‘Health gains and mastery’, ‘Learning’, ‘Affinity’, ‘Commitment’, and ‘Managing challenges’, they found incentives that fostered feelings of autonomy, competence and relatedness, leading to an increased motivation to exercise. Social support from coaches, structuring the physical environment with scheduled sessions, self-monitoring with resistance training log, and feedback based on heart rate monitor and fitness tests were the most valued BCTs.

**Conclusions:**

The results indicate the importance of finding incentives and creating an environment that fosters autonomy, competence and relatedness to motivate persons to exercise during oncological treatment. Some BCTs appear particularly useful and may be used by health professionals to increase patients’ motivation to exercise.

**Electronic supplementary material:**

The online version of this article (10.1007/s12529-019-09809-z) contains supplementary material, which is available to authorized users.

## Introduction

Exercise, defined as planned, structured and repetitive physical activity performed to maintain or increase physical fitness [1] has proven safe and beneficial during oncological treatment. Indeed, exercise may improve physical fitness and quality of life [2–5], reduce many disease and treatment-specific symptoms such as cancer-related fatigue [2, 4–6], anxiety [2, 3, 5] and several subsequent chronic diseases (e.g., diabetes, osteoporosis and cardiovascular diseases) in persons undergoing oncological treatment [2, 5]. Furthermore, epidemiological studies suggest that being physically active post-diagnosis may reduce the risk of cancer recurrence [7, 8] and improve the overall survival in cancer populations [8–10]. Based on current evidence, international physical activity guidelines for persons with cancer have been published. Adults undergoing oncological treatment such as chemotherapy, radiation and/or endocrine therapy are recommended to engage in cardiovascular endurance activity 150 min of moderate intensity or 75 min of vigorous intensity weekly in addition to two sessions of resistance exercise [2].

Not surprisingly, persons with cancer tend to reduce their level of physical activity after being diagnosed [11, 12] due to treatment side effects, lack of knowledge of exercise benefits during treatment and insufficient support from health professionals [5, 13]. The majority of persons undergoing oncological treatment do not meet the current international physical activity guidelines described above [5], suggesting that participating in exercise during this period is a real challenge. To facilitate exercise during oncological treatment, it is important to target psychological determinants such as readiness to change, self-efficacy for exercise and perceived behavioural control [14, 15]. Partly, overlapping these determinants, basic psychological needs such as autonomy (being the agent of one’s own actions), competence (being able to achieve challenging exercise tasks and reach goals) and relatedness (being connected to and valued by to others) may also influence exercise behaviour in cancer populations [16–19]. According to Self-Determination Theory (SDT) [20], the fulfilment of these three basic needs has a direct and positive influence on intrinsic motivation, which refers to the psychological drive to engage in a behaviour for its own sake and often involves feelings of satisfaction, enjoyment and personal accomplishment. When creating an environment that promotes these three basic needs, a person becomes intrinsically motivated and is more likely to sustain a behaviour change [20]. Thus, motivation is a central concept and using strategies that encourage the determinants mentioned above may lead to exercise behaviour change in cancer populations [14, 16–18].

Behaviour change support can be used to increase exercise motivation and facilitate exercise behaviour change in persons undergoing oncological treatment. It may include the use of specific behaviour change techniques (BCTs), defined as “observable, replicable, and irreducible components of an intervention designed to alter or redirect causal processes that regulate behaviour” (e.g. social support, goal-setting and self-monitoring) [21]. Previous quantitative reviews have reported positive effects from some BCTs on exercise behaviour during oncological treatment [22–24]. Thus, using such techniques is promising, but more detailed knowledge from an individual perspective is needed to inform health care and improve clinical practice. Indeed, no previous study within exercise oncology has explored motivation and behaviour change support [25, 26]. Furthermore, no study has focused on how specific BCTs are valued by persons exercising during oncological treatment. The aims of this study were therefore to explore the motivational experiences of exercise combined with behaviour change support, and to describe how specific BCTs were valued among persons exercising during oncological treatment.

## Methods

### Design

A concurrent triangulation study design was used, combining qualitative and quantitative data. This approach was chosen in order to obtain different but complementary data to answer the research questions. In this design, qualitative and quantitative data were collected and analysed independently but during the same timeframe and with equal priority. They are reported separately in the results and then merged in the discussion to give an overall interpretation of the findings [27].

### The Phys-Can Intervention Study

The present study was part of a multicentre randomized controlled trial, the Physical Training and Cancer (Phys-Can) intervention study, which is described in detail in the study protocol [28]. The Phys-Can intervention study aimed to compare the effects of low-to-moderate versus high-intensity exercise with or without additional behaviour change support on cancer-related fatigue and health-related quality of life in persons with cancer during and post-treatment. Six-hundred patients with newly diagnosed breast, colorectal or prostate cancer scheduled to undergo curative oncological treatment were consecutively recruited at University hospitals in three different regions of Sweden, located in the south, southeast and east of the country. Patients with cognitive dysfunction (e.g. dementia and serious mental illness), physical impairments or other diseases (e.g. cardiovascular and lung diseases) that could affect the ability to perform exercise were excluded. The participants were randomized to one of four intervention groups:1) low-to-moderate intensity exercise *with* additional behaviour change support, 2) low-to-moderate intensity exercise *without* additional behaviour change support, 3) high-intensity exercise *with* additional behaviour change support or 4) high intensity exercise *without* additional behaviour change support. The participants exercised during a period of six months, combining twice-weekly resistance training and cardiovascular endurance training. The resistance training was performed in group in a public gym and supervised by coaches (i.e. physiotherapists and personal trainers), while the endurance training was home-based.

To enhance adherence, all intervention groups were provided with specific BCTs, classified in accordance with the BCT taxonomy developed by Michie et al. [21], such as social support from coaches and peers, structuring the physical environment with scheduled resistance training sessions, and feedback with the use of heart rate monitor and fitness tests, including cardiopulmonary exercise tests (CPETs) and strength tests. Further, the two intervention groups *with *additional behaviour change support were provided with additional BCTs. This included self-monitoring with training logs, individual goal-setting, action planning with an initial interview on exercise habits and regular exercise planning, problem solving with regular coach-led reviews of motivation and long-term coping planning for exercise maintenance. A detailed description of how the BCTs were provided is presented in Table 1. The exercise programme started with a six-week familiarization period, providing the participants a gradual introduction to the exercise programme and the use of the different BCTs. The present study focused on the participants included in the intervention groups *with* additional behaviour change support i.e. the participants who were provided with all the specific BCTs described above.

**Table 1:** Description of the specific BCTs used in the Phys-Can intervention study

BCT	Component	Description
Social support	Coaches	The coaches provided the participants with encouragement and counselling during the training period. They gave information about why and how to exercise. They also provided practical help and guidance during the sessions
	Peers	The participants encouraged and assisted each other during the resistance training sessions. They also advised each other on how to perform exercise during oncological treatment
Structuring the physical environment	Scheduled resistance training sessions	The participants were provided with several cues to facilitate exercise: fixed times and a place to exercise, gym equipment
Feedback	Use of heart rate monitor	The participants were prompted to use a heart rate monitor during the endurance training sessions. It provided them with direct feedback on the intensity and duration of their performance
	Reviews of heart rate monitor files	The participants reviewed the heart rate monitor files monthly with the coaches and were provided with feedback on their performance
	PETs	The participants performed CPETs twice, i.e. at the beginning and at the end of the training period. They were informed about their results and progression
	Strength tests	The participants performed strength tests up to six times during the training period and were informed about their results and progression
Self-monitoring	Endurance training log Resistance training log	The participants monitored and recorded their training sessions in training logs. The logs also included notes and reflections on situations when the participants actually performed the session, when they did not and the subsequent consequences
Individual goal-setting		The participants were prompted to specify a behavioural goal for their performance in terms of exercise frequency, duration, intensity and/or type. Goal-setting was performed weekly at the beginning of the training period and more sparsely over time. The frequency was adjusted to individual needs, depending on how challenging it was for the participants to achieve their goals
Action planning	Initial interview on exercise habits	The participants were interviewed about their current exercise habits at the beginning of the training period as starting point for exercise planning
	Regular exercise planning	The participants were prompted to plan their endurance training sessions each time individual goal-setting was performed. The planning included exercise frequency, duration, intensity and type
Problem solving	Reviews to explore motivational issues	Concurrent with individual goal-setting and action planning, the participants analysed with the coaches their training logs and identified strategies to overcome barriers
	Coping planning for exercise maintenance	At the end of the training period, the participants made a long-term planning including strategies for overcoming barriers and increasing facilitators

*BCTs* behaviour change techniques, *Phys-Can* physical training and cancer, *CPETs* cardiopulmonary exercise tests

### Participants and Settings

All the participants in the Phys-Can intervention study, who were randomized to one of the two intervention groups *with *additional behaviour change support and had completed the familiarization period, were invited by mail to take part in the present study. In total, 229 of the 243 eligible participants (94%) agreed to participate in the quantitative part of the study. Among these participants, 20 were approached by telephone and 18 agreed to participate in the qualitative part of the study. The selection of these 18 participants was based on a maximum variation sampling strategy in order to capture as many different experiences as possible [29]. Dimensions of interest were age, gender, geographical location, level of education, occupation, cancer diagnosis, primary oncological treatment, previous exercise habits, exercise intervention group, attendance at the resistance training sessions and dropout of the exercise programme. The characteristics of the study participants are presented in Table 2.

**Table 2:** Characteristics of the study participants

	Questionnaire participants (*n* = 229)	Interview participants (*n* = 18)
Age (years), mean (SD)	59 (12)	63 (11)
Women, *n* (%)	178 (78)	9 (50)
Study site, *n* (%)		
Malmö/Lund	98 (43)	4 (22)
Linköping	31 (13)	3 (17)
Uppsala	100 (44)	11 (61)
Level of education, *n* (%)^a^		
Elementary school	22 (10)	2 (11)
High school	58 (25)	4 (22)
College/university	134 (58)	10 (56)
Other	9 (4)	2 (11)
Occupation, *n* (%)		
Working (full-time and part-time)	131 (57)	5 (28)
On sick leave (full-time and part-time)	82 (36)	5 (28)
Retired	94 (41)	9 (50)
Diagnosis, *n* (%)		
Breast cancer	175 (76)	8 (44)
Colorectal cancer	9 (4)	3 (17)
Prostate cancer	45 (20)	7 (39)
Primary oncological treatment, *n* (%)		
Chemotherapy	122 (53)	8 (44)
Radiation therapy	83 (36)	9 (50)
Endocrine therapy	24 (11)	1 (6)
Previous exercise habits, median (min-max)		
Resistance training, times/week^b^	0 (0–3)	0 (0–3)
Endurance training at moderate intensity, min/week^c^	180 (0–1000)	180 (0–550)
Endurance training at high intensity, min/week^d^	0 (0–600)	0 (0–600)
Exercise intervention group, *n* (%)		
High intensity	114 (50)	9 (50)
Low-to-moderate intensity	115 (50)	9 (50)
Attendance at resistance training sessions (max possible *n* = 54), median (min-max)	39 (7–55)	41.5 (7–51)
Dropout, *n* (%)	9 (4)	2 (11)

Missing values from the questionnaire participants:

^a^6 missing values

^b^36 missing values

^c^40 missing values

^d^49 missing values

The Phys-Can intervention study was approved by the Regional Ethical Review Board in Uppsala (Dnr 2014/249) and all participants gave informed written consent before participating. The participants were guaranteed confidentiality and were informed that they could withdraw from the study at any time.

### Data Collection

The qualitative and quantitative data were collected in parallel between June 2016 and November 2018, 0–9 months (of which 90% within three months) after the participants completed or dropped out of the exercise programme.

#### Interviews

An interview guide, consisting of six main areas with open-ended and follow-up questions, was developed to explore the participants’ motivational experiences of exercise combined with behaviour change support (Table 3). The questions were elaborated within each of the main areas to gain more detailed and rich descriptions from the participants. The interview guide covered questions about expectations and experiences of exercising in the Phys-Can intervention study, facilitators and barriers for exercising, as well as experiences of the specific BCTs and reasons for interrupting participation (optional). Responses were followed by probing questions such as “Could you tell me more about…?”, “Could you give me a concrete example?”. Individual semi-structured interviews were performed by the second author (MC) who was an experienced interviewer and was not involved in the Phys-Can intervention study. The first interview was used as a pilot interview to test and adjust the interview guide. The follow-up questions regarding the experiences of the specific BCTs were then modified, and no more major changes were made. The interviews took place at the interviewer’s office or, when this was not possible, by phone. The interviews lasted between 25 and 65 min and were audio-recorded. Data collection by interview continued until the authors judged that no new key themes emerged. The interviews were then transcribed verbatim.

**Table 3:** Main areas, open-ended and follow-up questions of the interview guide

1. Expectations before starting exercising What expectations did you have before starting exercising in the intervention?	
2. Experiences of exercising Could you tell me about your experiences of exercising in the intervention?	
3. Facilitators for exercising Describe what made it easier to exercise during the intervention. - What did help you to exercise? - What did motivate you to exercise?	
4. Barriers for exercising Describe what made it difficult to exercise during in the intervention. - Describe a situation where you did not manage to overcome obstacles. - Describe a situation where you did manage to overcome obstacles. - How did you manage to overcome obstacles?	
5. Experiences of the specific BCTs Could you tell me about your experiences of specific BCTs? - Which BCTs have been most useful to you for exercising? Why? - How did the BCTs help you to exercise? - Which BCT have been less useful to you for exercising? Why?	
6. Reasons for interrupting participation (optional) Could you tell me about the reasons for interrupting your participation? - What made you drop out of the exercise program? - What could have made you continue training in the intervention?	

BCTs behaviour change techniques

#### Questionnaire

A 19-item study-specific questionnaire was developed and designed to evaluate how the specific BCTs included in the exercise programme were valued. The questionnaire has been adjusted from an existing template for adults with rheumatoid arthritis [30]. The participants in the present study were asked to rate on a scale of 1 to 5 (1 = “Not at all valuable, 5 = “Very valuable”) the value of specific BCTs such as social support from coaches and peers, self-monitoring with training logs and goal-setting. The participants were also asked to rate on a scale of 1 to 5 (1 = “No, definitely not”, 5 = “Yes, definitely”) their intention to recommend the exercise programme in the Phys-Can intervention study to others with a similar condition. The questionnaire was sent by mail to the participants, including two reminders at three weeks and five weeks, respectively.

### Data Analysis

#### Interviews

A thematic analysis was performed as described by Braun and Clarke [31]. The analysis started with an inductive approach and assumed, in a later phase, a deductive profile inspired by the key motivational concepts in SDT [20]. The analysis was carried out in five steps. During the first four steps, the inductive approach was used. First, the transcribed interviews were checked against the recordings for accuracy. The transcribed interviews were then read several times to familiarize with the data, and initial ideas were noted down. Second, initial codes were generated through NVivo 11 software programme by systematically sorting the entire data set, and then tagging and naming extracts of interest. Third, the different codes were sorted into potential themes and subthemes, and a thematic map was produced. Fourth, the potential themes and subthemes were revised several times and were checked against each other and back against the original data set. Fifth, the deductive approach was used, and the themes and subthemes were further refined, defined, and named. The analysis was a back-and-forth procedure with several revisions. The first author (ASM) conducted the primary analysis, and all five authors (ASM, MC, SB, KN and ID) peer-reviewed the analysis seven times during the analysis process, over a time period of eight months. Subthemes and themes were defined and revised by the authors until a consensus was reached, and representative quotes were jointly selected. Furthermore, to ensure rigour and quality in the analysis, the authors followed the 15-point checklist developed by Braun and Clarke [31].

The authors had different professional backgrounds and expertise: the first author (ASM) was a physiotherapist and a PhD student. She was responsible for coaching two groups without additional behaviour change support in the Phys-Can intervention study. The second author (MC) was an oncology nurse, working as a researcher with expert knowledge in qualitative methods. The third and fourth authors (SB and KN) had extensive research experience and experience of the study context. Finally, the last author (ID) was a physiotherapist and a researcher with expert knowledge in behavioural medicine, exercise interventions and mixed methods. She was responsible for coaching two groups with additional behaviour change support in the Phys-Can intervention study.

#### Questionnaire

Response scales from the questionnaire were analysed by descriptive statistics (numbers, proportions, medians and interquartile ranges for the total group), using the Statistical Package for the Social Sciences (SPSS, v.24).

## Results

### Qualitative Results

**Fig. 1:**
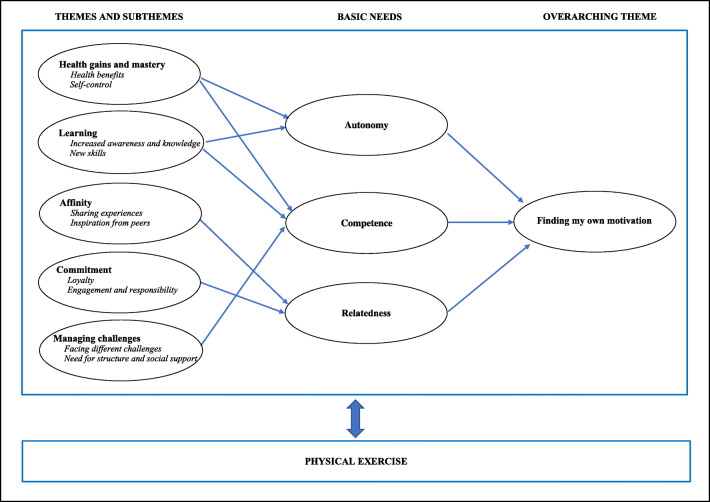
Integration of the qualitative findings and basic needs into the Self-Determination Theory (SDT) framework

An overarching theme ‘Finding my own motivation’ with five themes ‘Health gains and mastery’, ‘Learning’, ‘Affinity’, ‘Commitment’ and ‘Managing challenges’ were identified as the participants’ motivational experiences of exercise combined with behaviour change support during oncological treatment. The overarching theme can be understood as the result of an individual motivational process through the six-month exercise programme. The themes, detailed by 10 subthemes (in italics in the text), can be considered as a description of the incentives involved in this process that fostered the satisfaction of the three psychological basic needs described in SDT: autonomy, competence and relatedness (Fig. 1).

#### Theme 1: ‘Health Gains and Mastery’

The participants experienced both physical and psychological *health benefits *from the exercise programme as incentives to perform exercise. They felt more alert and described a feeling of improved mood and well-being after exercising. Increased vitality, improved muscular strength and fitness as well as better quality of sleep were also experienced.

“I felt that even just one exercise or something that raises my body temperature made it all feel better. After the (chemotherapy) drip, you become quite severely sensitive to the cold with severe tingling and numbness and sensitivities in the hands and feet, which was relieved just by getting a bit warmer.” #1

“I was generally much more alert when I forced myself even if I was tired and not feeling great, or felt ill, if I forced myself out on these walks it was nice. I felt much better. And then also with the resistance training, I remember that on a few occasions I really didn’t want to go but I forced myself to go to the gym and it felt much better afterwards…It really did.” #5

The experienced benefits were seen as rewards and led to the satisfaction of being able to promote their own health, fight the disease and counteract the treatment side effects, which gave the participants a feeling of *self-control *and competence. The feeling of self-control was also an important incentive to exercise. It gave the participants more energy and confidence to engage in other activities alongside the exercise programme. The participants therefore felt more motivated to continue exercising.

“So you don’t know what it would have been like if you hadn’t done the treatment but I was so motivated (to exercise). I sort of wanted to… spit in the face of the cancer in some way, like “you’re not going to get me” so to speak, sort of like that, so that I was really motivated.” #2

#### Theme 2: ‘Learning’

Participating in the exercise programme during oncological treatment led to an* increased awareness and knowledge* of exercise and its benefits, which was experienced as an important incentive to exercise.

“What struck me most was that it isn’t dangerous to train so hard, to lift something so heavy for your arms and legs. […]. I think that what gave me the most was that you could work out so hard, and that’s something I’ll never forget in my life, however, long that ends up being.” #4

“It was exciting for me to discover this. It didn’t really feel like much of an effort to bring your pulse up to around 110. It was like a fast, relatively fast walk. As soon as you started to jog a bit or cycled then you got up to 115, 120 or more and that’s when it quite suddenly got much harder. […] For me that was a little lesson, almost a lesson taught that to increase your fitness it has to be that hard, otherwise nothing much happens.” #12

It also allowed the participants to learn *new skills*. The participants learned more about how to exercise safely, about themselves, their body’s strengths and how to deal with treatment-related physical limitations. The participants also learned how to use specific BCTs including tools for self-monitoring and feedback such as training logs and heart rate monitor, making the training sessions more enjoyable and instructive. Other self-regulatory tools such as fitness tests, goal-setting and exercise planning were also mentioned as useful and sometimes even necessary to perform exercise.

“It (the heart rate monitor) meant that I managed (the exercise). And just having the monitor I thought felt like that I thought it was easier to do it. […] It was quite fun to look at it, how you increased your pulse and how long it stayed there, and it helped me to keep going until it beeped.” #7

The tools provided the participants with ways to manage and encourage self-reflection with a focus on personal achievement. The participants could see their progress and improvement, which increased their motivation and confidence in their ability to exercise. They felt more competent and became more autonomous in performing exercise.

“What was useful was that you could go back and look at your (notes in the resistance training log) […] It could be quite exciting to see if you scrolled back through and thought that there the weights weren’t actually very heavy but at the time I thought it was really really hard and now I am lifting quite a lot more weight and that’s how you… you monitor yourself. You can see your development in the (resistance training log)." #7

“Really fantastic (fitness tests), I mean really. And then it was also like a, I mean you saw that you had become stronger, so it was like a sort of positive sort of motivation to continue.” #11

#### Theme 3: ‘Affinity’

Exercising in a group with other persons diagnosed with cancer was another important incentive to exercise and gave insights that the participants were not alone in facing the challenges of the disease and the treatment. They* shared their experiences *about how to deal with treatment side effects, their feelings and concerns about the disease, and shared tips and advice on how to exercise. In other words, the group was an important source of information.

“This here with treatments in particular and this stuff, of course we have had somewhat different treatments for prostate and for breast cancer, what they experience how they, what their treatments are and well, what the side effects are for them. That you are not alone in this, but you know that the others have similar side effects. You don’t need to go and worry about it, it’s just something you get. So, you get a lot of information in that way which is good.” #9

The group was also an important source of *inspiration*, allowing the participants to compare how they dealt with the disease and how they performed exercise. The participants felt connected to each other as a team, caring for each other and working towards the same goal. This group cohesiveness gave the participants a feeling of security and provided them with a sense of improved mental strength and increased motivation to exercise, creating a favourable climate for exercise enjoyment.

“It was sort of lovely to see, sometimes when I myself was very down and I saw someone else who is in almost the same phase as I am, more alert and can talk and laugh and the like. So, it gave hope and joy and strength.” #5

“It turned out to be a nice group all in all, it was fun going there and meeting others and talking with them about this, the problems you’ve had or not had.” #16

#### Theme 4: ‘Commitment’

The participants had a strong sense of commitment. Their *loyalty* to the coaches and the group was an important incentive to go to the gym and to exercise.

“Then it’s definitely made things easier I think, the coaches …you don’t want to disappoint them if you understand what I mean. They’re there for us and so we have to turn up for them, a bit like that is how it felt. […] No but that’s what’s made it easier I think, that their enthusiasm was like, and their presence and commitment I think felt like you couldn’t just skip this sort of... It became some sort of inner spirit there that says “nah, just gotta do this now”.” #12

“If you’re in a team you never miss a match and I’ve never done that so or I must have been very sick in that case if I did do that, or I’ve never missed you know, because if you’re in a team you just do it. It’s important to me anyway.” #15

The participants also had a strong sense of *engagement and responsibility*. Participating in a research study was taken seriously and was an incentive to complete the exercise programme. It was sometimes seen as an obligation or a duty, but it gave the participants a sense of purpose and a feeling of being part of something bigger.

“Yes, it was probably so that I took this on as a, what can I say, almost like a mission. I’d decided to be part of this (study), so I have to do this thing here. And then it was like nothing, for me it was never anything, I never questioned that I would do it because, if I’ve said I’m going to do it then that’s what I’m going to do.” #7

#### Theme 5: ‘Managing Challenges’

The participants had to *face different challenges *during their oncological treatment that affected their ability to exercise. Fear of movement, fear of infection and injury were mentioned as barriers to exercise. They also faced symptoms due to the treatment such as numbness and tingling, fast heart rate, tiredness, pain and cognitive impairments, which sometimes felt insurmountable and made exercise difficult to perform. Changes in appearance (e.g. hair loss), urinary symptoms or bowel disturbances were other treatment side effects that made the participants uncomfortable and were pointed out as barriers to exercise.

“I thought about it a lot, like, they say a lot about how you have to be beware of infections, you should be careful and so on. And I didn’t go to the cinema, I didn’t travel by bus, I wasn’t in town, but I don’t go there willingly anyway. So, I was a bit careful, and then I felt anyway “I see, so I’m supposed to work out with people who may be going around coughing. How is that going to turn out?”” #2

“For me it was a bit difficult because my pulse went up incredibly high from the chemotherapy, so it caused a lot of anxiety and worry” #11

Practical barriers such as long distance and travel to the public gym as well as lack of time were mentioned by the participants. Those who worked expressed difficulties in attending the resistance training sessions during working hours. Furthermore, the participants who experienced a lack of enjoyment in exercising found it hard to motivate themselves to complete the programme.

“Then it was like this, I felt after a while that I hadn’t been at training and felt like “no, now I feel like I’m not getting anything out of it”. Initially my idea was that I wanted to be involved in this research to help with the results and be able to help others but then that’s how it turned out but I’m getting nothing out of it, and this really isn’t my thing, to train in this way. Instead of this what I do is that I walk, maybe start cycling, do things here that make me feel the best. Then my motivation disappeared entirely.” #5

However, the *structure and social support *provided by the coaches and other participants was an important incentive to exercise, and helped to overcome and manage these challenges, giving the participants a feeling of competence. Scheduled training sessions helped the participants to bring structure into their everyday lives and was seen as a distraction from the disease, while exercising in a public gym helped them to feel normal and healthy. Being supervised by experienced coaches was also an important aspect, providing the participants with a feeling of safety, guidance and positive feedback on their exercise performance.

“You know I think that this has been great and above all that you had something that you were supposed to do, that there were appointments because otherwise it could easily have been like this “no but today I’m not feeling so great” but you knew that you had an appointment on Mondays at 9:30 am when you had to get up and eat reasonably well so that I would be there by 9:30 am, otherwise perhaps you would just sit on the couch all day and “I’ll train another day “, so I think having those fixed times has been quite good” #6

Using social support from family and friends (e.g. exercising together and being encouraged) was another key to overcoming and managing the challenges the participants faced. The participants often managed to exercise more than they expected, which increased their motivation and confidence in their ability to participate in challenging tasks.

“This endurance training, I’ve had my husband who has encouraged me as well. So that when he has seen that I was getting a bit tired or a bit so-so, he has asked me like “how much time do we have left this week?”, and then he has said “right well let’s get cracking then” sort of. So that he has encouraged me in fact and that feels good.” #8

### Quantitative Results

The quantitative results from the questionnaire demonstrated that all the specific BCTs included in the exercise programme were highly valued by the participants with medians ranging from 3 to 5 on a 1–5 scale (1= “Not at all valuable” and 5 = “Very valuable”). Detailed results are presented in Table 4.

**Table 4:** Results from the questionnaire on the value of specific BCTs and intention to recommend the program (*n* = 229)

BCT (component)	*n*^a^	Median	Q1–Q3
Supervised resistance training^b^			
Action planning (initial interview on exercise habits)	221	4	4–5
Social support (coaches)	226	5	5–5
Social support (peers)	222	4	3–5
Structuring the physical environment (scheduled sessions)	225	5	4–5
Feedback (strength tests)	222	5	4–5
Self-monitoring (resistance training log)	220	5	4–5
Problem solving (coping planning for exercise maintenance)	210	4	4–5
Home-based endurance training^b^			
Action planning (initial interview on exercise habits)	214	4	4–5
Action planning (regular exercise planning)	208	4	3–5
Social support (coaches)	215	5	4–5
Social support (peers)	202	3	2–4
Individual goal-setting	205	4	3–5
Feedback (CPETs)	211	5	4–5
Feedback (use of heart rate monitor)	219	5	4–5
Feedback (reviews of heart rate monitor files)	203	4	4–5
Self-monitoring (endurance training log)	214	4	3–5
Problem solving (reviews to explore motivational issues)	208	4	4–5
Problem solving (coping planning for exercise maintenance)	198	4	4–5
Intention to recommend the program to others with a similar condition^c^	223	5	5–5

^a^Numbers vary due to internal attrition

^b^Response to the 1–5 rating scale: 1 = Not at all valuable and 5 = Very valuable

^c^Response to the 1–5 rating scale: 1 = No, definitely not and 5 = Yes, definitely

*BCTs* behaviour change techniques, *CPETs* cardiopulmonary exercise tests

For supervised resistance training, the most valued specific BCTs were social support from coaches, structuring the physical environment with scheduled sessions, self-monitoring with resistance training log and feedback with strength tests - all rated with a median of 5 (“Very valuable”). For home-based endurance training, the most valued specific BCTs were social support from coaches, feedback with the use of heart rate monitor and CPETs, all rated with a median of 5 (“Very valuable”). Intention to recommend the programme to others with a similar condition was also rated with a median of 5.

## Discussion

The present study used a mixed methods approach to explore the motivational experiences of exercise combined with behaviour change support and to describe how specific BCTs were valued among persons undergoing oncological treatment. The study results indicate that the participants underwent a motivational process through a six-month exercise programme during oncological treatment. They described their incentives to exercise through experiencing health gains and mastery, gaining increased awareness and knowledge, learning new skills, feeling affinity with peers, feeling commitment to the coaches and the study, and managing the challenges they faced during oncological treatment. These incentives in turn fostered autonomy, competence and relatedness, resulting in finding their own motivation. Furthermore, social support from coaches, structuring the physical environment, and the use of tools for self- monitoring and feedback were valued as the most salient BCTs by the participants.

The participants described how they experienced ‘Health gains and mastery’ as incentives to exercise, giving them a sense of autonomy and competence. This finding is in line with a review of qualitative studies among breast cancer survivors describing how exercise increased empowerment, well-being and energy [25]. ‘Learning’ was also described as an important aspect for gaining exercise motivation through an increased sense of autonomy and competence. Using specific BCTs such as self-monitoring and tools for evaluating physical improvements gave the participants feedback on exercise behaviour and increased their confidence in their ability to exercise. This is also highlighted by the results from the questionnaire, where the use of resistance training log, heart rate monitor and fitness tests were highly valued. These findings are in line with reviews of randomized controlled trials among different cancer populations that have described self-monitoring as a promising BCT [22, 23]. The participants also described the importance of feeling ‘Affinity’ with their peers and ‘Commitment’ to the coaches and the study, which gave them a feeling of security and relatedness and helped them find their own motivation to exercise during oncological treatment. Here, the results from the interviews and the questionnaire are somewhat contradictory. While the participants in the interviews emphasized the importance of social support from peers, the results from the questionnaire are not as clear, with a lower value for this specific BCT than the others. One possible explanation is the fact that peer support was an important aspect for the study participants but not as crucial as the other specific BCTs were for performing exercise, especially regarding endurance training. Indeed, endurance training was home-based, i.e. the participants performed the sessions on their own without direct support from peers. The importance of peer support in patients undergoing oncological treatment has been emphasized in previous qualitative studies [25, 26, 32]. These studies support the implementation of group-based exercise, highlighting the social benefits of group settings by increasing “the participants’ morale” and their motivation to exercise. Finally, the participants expressed a need for structure and social support for ‘Managing challenges’ faced during oncological treatment, giving them a feeling of competence and increased motivation to exercise. They described how scheduled training sessions helped them to bring structure into their everyday lives and distract them from the disease while being supervised by experienced coaches provided them with a feeling of safety, guidance and positive feedback on their exercise performance. The importance of structuring the physical environment with scheduled resistance training sessions and using social support from coaches is also reflected in the results from the questionnaire, as these two BCTs were highly valued. Similarly, Midtgaard et al. [26] described in their review of qualitative studies among cancer survivors how planned exercise gave the participants “something to look forward to” and “resembled going to work”, which provided a sense of control over their life and the disease. Furthermore, several qualitative reviews [25, 26] also highlighted the importance of skilled and knowledgeable coaches to feeling safe to exercise during oncological treatment. Social support from family and friends was another essential aspect highlighted in the interviews. Exercising together with and being encouraged by family and friends helped the participants to manage to exercise more than they expected, which increased their confidence and motivation to exercise. In contrast to our results, previous qualitative studies have reported lack of social support from family and friends [25, 33–35]. These studies described pressure from family and friends as a barrier to participating in exercise during oncological treatment, which could be explained by fear of exercise being harmful during oncological treatment [25, 34]. In our study, this issue was not encountered, possibly because family and friends were involved during the process of behaviour change support (e.g. during regular follow-ups for exercise planning where the participant’s motivational issues and social support were explored). Indeed, involving family and friends in exercise interventions has been suggested as a positive approach, allowing them to have a better understanding of the interventions and thereby providing the participants with a greater support [36].

In other words, our results reflect the importance of creating a favourable environment for performing exercise during oncological treatment, fostering feelings of autonomy, competence and relatedness. This is in line with the SDT theoretical framework [20] and demonstrates that intrinsic motivation, corresponding to the overarching theme ‘Finding my own motivation’, can be enhanced through an exercise programme combining behaviour change support, supervised resistance group training in a public gym and home-based endurance training.

Our results may contribute to the development of clinical practice. Health professionals are in a unique position to promote patients’ exercise behaviour during oncological treatment, but few recommend it due to a lack of knowledge in exercise counselling [5]. While the current international physical activity guidelines for persons with cancer provide health professionals with a frame for general recommendations, there is a need for increased knowledge about how to implement these guidelines in practice. Our results provide health professionals with such information and concrete examples of which specific BCTs to use and how to use them as helpful tools to change exercise behaviour in patients undergoing oncological treatment. Furthermore, our results could also be used in training contexts that should be provided to health professionals at basic levels and in further education. However, it is important to consider that the study participants agreed to participate in a demanding exercise intervention for six months and thus were per definition motivated enough to make this decision. In clinical practice, there is a more heterogeneous patient population with all levels of motivation and different exercise habits. It should be pointed out that the results in the present study may not be applicable to all patients and that each patient should be provided with a tailored exercise programme based on physical, psychological and contextual aspects.

The described methodology has strengths and limitations. Several strategies were used to ensure trustworthiness of the results [37]. First, to address credibility, a maximum variation sampling strategy was used in the qualitative part of this study, resulting in a selection of participants with a wide range of variation on dimensions of interest. This sampling strategy was used to maximize the range of information uncovered by capturing many and various experiences in order to give a rich, in-depth picture of these experiences [29]. Another strength is the data triangulation combining interview and questionnaire data, providing both in-depth descriptions and an overall group-level description. One possible limitation is the risk of memory bias among the participants when describing their experiences and answering the questionnaire. However, the majority of the data were collected within three months after the participants completed or dropped out of the exercise programme. Second, to ensure dependability, the research process of the study has been reported in detail, using the checklist developed by Braun and Clarke [31] on how to perform thematic analysis, and also the Consolidated criteria for reporting qualitative research (COREQ) [38], which enables readers to follow the research process and repeat the study. Third, to ensure confirmability, frequent discussions with authors having different professional backgrounds and expertise enabled reflections on alternative approaches and interpretations of the collected data. It was also a way to ensure that the interpretations and findings were clearly derived from the data and not from the authors’ preferences and preunderstanding. Furthermore, the theoretical model based on SDT [20] served as a framework for the interpretation, enhancing objectivity and preventing the authors’ preunderstanding from influencing the results. Finally, to address transferability, details of the setting and participants were provided, allowing a comparison of the study’s context to other possible contexts. However, as described above, we cannot claim that our findings can be applied to other populations and situations but by providing such details, readers may evaluate for which target groups the study provides valuable information and can thus judge its transferability [39]. Moreover, confidence in this study’s transferability may be strengthened by its mixed methods design, through the maximum variation sampling used in the qualitative part and the large sample size used in the quantitative part [40].

## Conclusions

The results of the present study indicate the importance of finding incentives and creating an environment that fosters feelings of autonomy, competence and relatedness to facilitate exercise during oncological treatment. Our results also highlight the importance of using social support, structuring the physical environment, and using tools for self- monitoring and feedback to encourage persons to exercise during oncological treatment. These specific BCTs can be implemented and used by health professionals to increase patients’ intrinsic motivation to exercise. However, further research is needed to evaluate how to individualize exercise during oncological treatment among patients with low motivation and limited experience of exercise.

## Electronic supplementary material

ESM 1 (PDF 312 kb)

## Abbreviations

BCTs: behaviour change techniques
CPETs: cardiopulmonary exercise tests
Phys-Can: physical training and cancer
SDT: self-determination theory
